# Female unmarried adolescents’ knowledge on selected reproductive health issues in two low performing areas of Bangladesh: an evaluation study

**DOI:** 10.1186/s12889-015-2597-1

**Published:** 2015-12-21

**Authors:** Humayun Kabir, Nirod Chandra Saha, Rukhsana Gazi

**Affiliations:** Centre for Equity and Health Systems, International Centre for Diarrhoeal Disease Research, Bangladesh (icddr,b), 68 Shaheed Tajuddin Ahmed Sarani, Dhaka, Dhaka 1212 Bangladesh; Centre for Vaccine Sciences, International Centre for Diarrhoeal Disease Research, Bangladesh (icddr,b), Dhaka, Bangladesh

**Keywords:** Female unmarried adolescent, Knowledge, FP methods, HIV/AIDS, STI, Reproductive health

## Abstract

**Background:**

In Bangladesh, 24 % of the total populations are adolescents. Twelve months intervention was implemented under Demand-Based Reproductive Health Commodity Project (DBRHCP) in two low performing areas: rural Sub-district Nabiganj (population 323,357) and an urban slum in Dhaka city (population 141,912). We evaluated the changes in knowledge of female unmarried adolescents on selected reproductive health issues over the project period in two low performing areas of Bangladesh.

**Methods:**

A pre-post study design was adopted. Under DBRHCP, interventions were focused on training of government service providers, disseminating behaviour change materials within the targeted communities, and employing community-based health promoters (Community Support Group and Peer Promoters) to foster linkages between the community and providers. All households were enumerated. A baseline survey was conducted during November 2006 to March 2007 and an end-line survey was conducted during November 2008 to March 2009. Eight hundred female unmarried adolescents (12–19 years) were selected independently for each survey from each study area through systematic random sampling, capturing changes over the 12 months intervention period. Data was analyzed using SPSS. A chi-square test was used to assess the changes in knowledge between baseline and end-line among the female unmarried adolescents.

**Results:**

Female unmarried adolescents had significantly increased knowledge at the end-line about measures to be taken during menstruation like: using clean and dry cloths. Overall, two-third of female unmarried adolescents knew about Family Planning (FP) methods in both study areas but had significantly increased knowledge on injectables and condoms at the end-line. Overall knowledge on Human Immunodeficiency Virus/Acquired Immune Deficiency Syndrome (HIV/AIDs) was markedly different in the urban and rural areas, but a significantly higher proportion of female unmarried adolescents knew about HIV/AIDs from relatives and school curricula, and had increased knowledge about mode of transmission of HIV/AIDs, like: receiving blood from an HIV infected person and using a HIV infected needle or syringe, at the end-line. A significantly higher proportion of female unmarried adolescents reported sexually transmitted infection (STI) related symptoms at the end-line compared to baseline. Overall variation in knowledge about Government healthcare facilities, Non-Government Organization (NGO) healthcare facilities and private healthcare facilities was found in both study areas, but awareness was increased about the type of healthcare facilities at the end-line.

**Conclusion:**

The improvement of the female unmarried adolescents’ knowledge on selected Reproductive Health (RH) issues suggest that the interventions affected RH related knowledge reported in the study. These interventions can be adapted in the health service delivery system to enhance people’s knowledge on RH issues to achieve RH for adolescents.

## Background

Globally, one-fifth of the total population are adolescents; among all adolescents, 85 % live in developing countries [1]. Ensuring the healthy reproductive health status of adolescents, particularly in developing countries, is a growing concern [2]. Adolescence is a crucial and vulnerable period when physical and mental changes take place and adolescents feel stressed due to these transitions [3]. A review in 2011, based on demographic health surveys (DHS) conducted in 11 countries (Cambodia, Indonesia, Marshall Islands, Nauru, Papua New Guinea, Philippines, Samoa, Solomon Islands, Timor-Leste, Tuvalu and Vietnam), reported that adolescents have poor knowledge and less access to information and services on reproductive health [4, 5]. The majority of young people in the Asia-Pacific region are not able to receive reproductive health services. They are vulnerable to reproductive health risks and sexually transmitted infections, including HIV. Their human rights are violated due to this denial of reproductive health services and education. The fifth Asia Pacific Conference on Reproductive and Sexual Health and Rights, held in Beijing, China, 2009 declared the need to improve the sexual and reproductive health and rights of young people [6].

Bangladesh’s Strategic Plan for Health, Population and Nutrition Sector Development Programme 2011–2016, has prioritized safe motherhood, family planning, menstrual regulation, care for post-abortion complications, and management of STIs, with specific guidelines laid out in the National Reproductive Health Strategy [7]. Both the Directorate General of Health Services and Directorate General of Family Planning provide reproductive health services through their maternal, neo-natal, and child health (MNCH) programs, including reproductive, sexual health and family planning [7]. Married adolescent women are the main beneficiaries for these services. In Bangladesh, unmarried female adolescents receive limited attention for reproductive healthcare issues by these existing programs.

Unmarried adolescents are not adequately informed of their physical well-being, health, and body-systems particularly when compared to their married adolescent peers [8]. The current information and services that are available in Bangladesh are not specific to adolescents, and the quality of such information and services is often poor or inappropriate for this age-group [8]. Unmarried adolescents often feel discomfort to discuss reproductive health concerns with parents, healthcare providers, and educators [9]. Parents, healthcare providers, and educators also do not provide complete, accurate age-specific reproductive health information to unmarried adolescents [10]. In 2000, a study was conducted by Family Planning Association of Bangladesh. The study found that a large proportion of adolescents had inadequate knowledge on problems related to menstruation, gonorrhea, syphilis, HIV/AIDs, and the availability of treatment or facilities for treatment for STIs [11]. Findings from a study in Bangladesh reported that only 13–14 % of the study adolescents knew about syphilis and gonorrhea, while about one-half of adolescents could not identify a single STI symptom and more than half could not describe a mode of transmission for an STI [11].

In 2005, the National Institute of Population Research and Training (NIPORT) under the Ministry of Health and Family Welfare (MoHFW) of the Government of Bangladesh implemented the 3 year (2005–2008) DBRHCP [12]. Under DBRHCP, interventions were focused on the training of government service providers, disseminating behaviour change materials within the targeted communities, and employing community-based health promoters (Community Support Group and Peer Promoters) to foster linkages between the community and providers. This paper is presenting one part of the findings from the evaluation of this large project. The specific objective of the present study was to assess changes in knowledge among female unmarried adolescents on selected reproductive health issues in two low performing areas of Bangladesh over the intervention period (12 months).

### Objective

We evaluated the changes in knowledge of female unmarried adolescents about selected reproductive health issues like: measures to be taken during menstruation, knowledge on family planning methods, HIV/AIDs, mode of transmission of HIV/AIDs including source of information, knowledge of STIs, reported STI symptoms, and health care facilities.

## Methods

### Study design and population

The study was implemented in two low performing areas: Nabiganj Upazila (sub-district) in the Sylhet division and four wards of Dhaka urban slum under Dhaka division of Bangladesh. The Ministry of Health and Family Welfare (MoHFW), Government of Bangladesh (GoB), ranked districts and sub-districts based on four indicators using health and FP services utilization: percentage of pregnant women, crude birth rate, contraceptive-use rate, and coverage of two doses of vitamin A capsules. The MoHFW found that most low performing districts/sub-districts were located in Sylhet and Chittagong divisions. Nabiganj is a low performing sub-district under Sylhet division, and the slums in Dhaka city are also low performing areas. Therefore we selected Nabiganj and four slum areas of Dhaka City Corporation purposively for this study based on the above mentioned criteria. Both the areas are known as a low-performing areas with low RH indicators [13, 14]. The Population Council, Research Training and Management International (RTM International), John Snow International/Deliver Bangladesh and icddr,b (International Centre for Diarrhoeal Disease Research, Bangladesh) were involved in implementation of this project. icddr,b evaluated the intervention activities through a baseline and end-line survey.

DBRHCP was an operation research (OR) project to improve the knowledge, and practice of targeted populations (married and unmarried) in order to meet their RH needs. Needs assessments were done at the beginning of the project to design the intervention strategies. RTM International and Population Council were involved for intervention activities in the project areas. They conducted the Behavior Change Communication (BCC) intervention for female unmarried adolescents. Under the BCC intervention, the activities were: i) formation of Community Support Groups (CSGs), ii) formation of peer promoters, iii) stage dramas, interactive jari gan and video shows. The objective of these activities was to increase awareness about RH and availability of RH services.

#### Community support group

Each CSG was consisted approximately 7–8 members, including elected representatives of the local government, field workers, religious leaders, teachers, social activists, unmarried females, and representatives of both rich and poor community members. A total 36 CSGs were formed. CSG members played an important role in improving the physical infrastructure of healthcare facilities as well as in cooperating with peer promoters to motivate unmarried females to seek services from trained service providers.

#### Peer promoters

Married, unmarried, male and female-were selected as peer promoters to assist the field workers in their routine activities. Peer promoters organized group and one-to-one sessions at the community level to improve knowledge on RH and increase awareness about availability of services in healthcare facilities and RH services. The peer promoters played a bridging role between community members and the health facilities.

#### Stage dramas, interactive jari gan and video shows

The local cultural groups staged a total of 65 dramas where approximately 300 community members attended each time. Total 65 video shows were organized on reproductive health issues with the help of District Information Office and Department of Mass Communication. These shows were organized in the evening. The contents of the video show included safe motherhood, family planning, child health, gender, male involvement, services available in the service delivery center, etc. Ten jari gan events were held on reproductive health issues in the project areas. With the assistance of CSG members and field workers, a local cultural group organized a drama to increase awareness of family planning, reproductive health and maternal health care services.

This paper only highlights the changes of knowledge on selected RH issues among female unmarried adolescents over the intervention period as a part of evaluation of the large project.

Inclusion criteria required participants to be female, age 12–19 years and currently female unmarried adolescents. We excluded respondents if more than one female unmarried adolescent fulfilled the criteria from the same household. Female unmarried adolescents who were not residents in the area were also excluded. In households where potential participants resided but who were absent on the first attempted visit, up to three subsequent attempts were made to reach absentees.

At the initial stage of the study all households in the study areas were enumerated to collect basic information regarding age, sex, marital status, socio-economic condition, and other similar variables in order to develop the sampling frame under DBRHCP. According to this enumeration, there were 54,116 households in Nabiganj and 29,904 households in urban slum area of Dhaka city. After household enumeration, a baseline survey was conducted among female unmarried adolescents aged 12–19 years during November 2006 to March 2007. An end-line survey was done during November 2008 to March 2009. The sample size was 800 per site. The sample size was estimated based on a 95 % confidence with 90 % power and a 25 % non-response rate. The sample size calculation was based on prior research conducted by icddr,b in Abhoynagar and Mirsarai, which estimated that approximately 5 % of adolescents from the rural area utilized services from government facilities or NGOs [15]. A total 800 female unmarried adolescents were selected independently for baseline and end-line survey from each study area by simple random sampling. The sample was drawn from the household enumeration list.

### Survey measures and data collection

Experienced female interviewers who had previously worked in different public health research studies were recruited to serve as interviewers for this study. Selected interviewers received 1 month extensive training on adolescent health, reproductive health, menstruation, family planning methods, HIV and STIs, existing health service facilities and availability of services, and interview techniques. Trained interviewers interviewed female unmarried adolescents using a structured questionnaire. Each day after returning from the field, the interviewers crosschecked the completed questionnaires identify potential errors/mistakes. The field supervisors reviewed each of the questionnaires and conducted regular spot-checking to maintain data quality. An experienced field research manager coordinated the overall field activities.

The structured questionnaire was adopted from previous icddr,b studies, which also explored RH related knowledge among female unmarried adolescents. Prior to survey implementation, the study questionnaire was field tested and followed with a debriefing session to identify any errors or necessary corrections to the questionnaire. The questionnaire included items to assess knowledge and perception of menstruation, knowledge of family planning methods, perceptions of mode of transmission of HIV/AIDs including source of information, knowledge of STIs and reported STI symptoms, and utilization of health care facilities. STI-related symptoms included burning during urination, genital ulcer/sores, and excessive bleeding. These questions were prompted using the following two questions: “Have you heard about sexually transmitted diseases, what are those?” and “Have you experienced any of the following STI symptoms in the last 1 year?”.

### Ethical consideration

Ethical approval for the study was obtained from the Ethical Review Committee (ERC) of icddr,b. The parents of female unmarried adolescents were asked for consent to allow their daughters to participate in the study. The study team received informed parental consent for all study participants including those under the age of 16. Once parental consent was obtained, the study team then approached the study participants. The parental non-response rate was 7 % in rural areas and 10 % in urban areas, but had already been corrected for in overall sample size estimation. None of the potential participants refused to participate. Data collection was anonymous and privacy was maintained during data collection. All interviews were conducted in a private corner/place of the household. To ensure confidentiality and anonymity, only identification numbers were used during data collection.

### Data analysis

Descriptive analysis was conducted to estimate distributions of relevant characteristics of the sampled populations. A Bivariate analysis was done to compare baseline and end-line with respect to participant age, education, knowledge and perception of menstruation, knowledge of family planning methods, perceptions of mode of transmission of HIV/AIDs including source of information, knowledge of reported STI diseases and symptoms, and utilization of health care facilities. Proportions, 95 % confidence intervals (CIs), and *p* values were calculated for selected variables. SPSS version 10.0 (IBM Corp, Armonk, NY) was used for all statistical analysis. Chi square tests were used to see the difference in knowledge on selected RH issues between baseline and end-line among female unmarried adolescents.

## Results

Table 1 shows that the age distribution of female unmarried adolescents was similar in the baseline and the end-line survey. In both the baseline and end-line, the proportion of female unmarried adolescents aged 12–14 was highest in the urban slum areas (44 % in baseline vs. 52 % in end-line) and the proportion of female unmarried adolescents aged 15–17 was highest in rural Nabiganj (50 vs. 49 %). In both urban and rural areas, a slightly lower proportion of female unmarried adolescents had never been enrolled in school at the end-line survey (8 %) compared to baseline (10 %). In both the baseline and end-line surveys, an education level of above the 5^th^ grade was almost similar in rural area (53 vs. 54 %) and urban slum area (43 vs. 41 %) respectively.

**Table 1:** Profile of unmarried female adolescents, by study areas

Age in years	Urban Slums Dhaka		Rural Nabiganj	
	Baseline *n* = 800	End-line *n* = 808	Baseline *n* = 833	End-line *n* = 824
12–14 years	44 %	52 %	27 %	31 %
15–17 years	39 %	35 %	50 %	49 %
18–19 years	17 %	13 %	23 %	20 %
Education				
Never enrolled	10 %	8 %	10 %	8 %
1–5 years	57 %	59 %	47 %	46 %
6+ years	43 %	41 %	53 %	54 %

Table 2 shows that female unmarried adolescent were significantly less inclined to use old cloths during menstruation at the end-line survey compared to baseline in urban (77 vs. 49 %; *p* < 0.001) and rural (60 vs. 54 %; *p* < 0.01) areas, respectively. In the end-line survey, knowledge about use clean dry cloth during menstruation improved significantly by female unmarried adolescents in urban (24 vs. 49 %; *p* < 0.001) and rural area (33 vs. 47 %; *p* < 0.001). Both in the baseline and end-line survey, female unmarried adolescent’s knowledge about use sanitary pad/cotton was similar in urban area (34 %), whereas slightly higher in rural area at the end-line survey compared to baseline (25 vs. 28 %). In rural area, knowledge improved significantly about use antiseptic liquid/hot water for washing during menstruation by female unmarried adolescents at the end-line compared to baseline (14 vs. 30 %; *p* < 0.001) and not significant in urban area (33 vs. 34 %). In rural area, knowledge about abstain from eating meat/fish/outside home during menstruation decreased significantly by female unmarried adolescents at the end-line compared to base line (14 vs. 6 %; *p* < 0.001) and significantly increased in urban slum area (4 vs. 11 %; *p* < 0.001).

**Table 2:** Knowledge about measures to be taken during menstruation

Measures taken	Urban Slums Dhaka			Rural Nabiganj		
	Baseline *n* = 800	End-line *n* = 808	*P*-value	Baseline *n* = 833	End-line *n* = 824	*P*-value
Use old cloths	77 %	49 %	***	60 %	54 %	**
Use clean dry cloth	24 %	49 %	***	33 %	47 %	***
Use sanitary pad/cotton	34 %	34 %	NS	25 %	28 %	NS
Use antiseptic liquid/hot water for washing	33 %	34 %	NS	14 %	30 %	***
Abstain from eating meat/fish/ outside home	4 %	11 %	***	14 %	6 %	***
Abstain from religious activities	11 %	13 %	NS	16 %	9 %	***
Take nutritious/good food	1 %	6 %	***	3 %	2 %	NS

Multiple responses; *p < 0.05; **p < 0.01; ***p < 0.001; *NS* Not significant

More than 60 % of female unmarried adolescents had heard about contraceptive methods both in the baseline and end-line survey, but this was not significant (Table 3). Their knowledge on the oral contraceptive pill was universal in both study areas (more than 90 %). In end-line survey, a significantly higher proportion of female unmarried adolescents knew about injectable contraceptives compared to baseline survey in urban area (42 vs. 59 %; *p* < 0.001) and rural area (55 vs. 61 %; *p* < 0.01) respectively. In the urban area, a significantly higher proportion of female unmarried adolescents in end-line survey had heard about condom compared to baseline (28 vs. 38 %; *p* < 0.001). Knowledge about long-term contraceptive methods (IUD, Implant and Sterilization) decreased significantly by female unmarried adolescents at the end-line compared to baseline in both areas.

**Table 3:** Knowledge on family planning methods

Knowledge on contraception for family planning	Urban Slums Dhaka			Rural Nabiganj		
	Baseline *n* = 800	End-line *n* = 808	*P*-value	Baseline *n* = 833	End-line *n* = 824	*P*-value
Knew about FP methods	61 %	62 %	NS	66 %	63 %	NS
Condom	28 %	38 %	***	19 %	18 %	NS
Pill	98 %	98 %	NS	95 %	98 %	***
Injection	42 %	59 %	***	55 %	61 %	**
IUD	2 %	1 %	NS	8 %	5 %	**
Implant/Norplant	6 %	5 %	NS	22 %	10 %	***
Female Sterilization	3 %	8 %	***	17 %	8 %	***
Male Sterilization	0.2 %	4 %	***	2 %	1 %	NS

Multiple responses; *p < 0.05; **p < 0.01; ***p < 0.001; *NS* Not significant

Table 4 shows that a significantly higher proportion of female unmarried adolescents in the end-line survey (80 %) had heard about HIV/AIDS compared to baseline (73 %; *p* < 0.001) in the urban area. Television was the most common source of information about HIV/AIDs in the urban (95 %) and rural area (86 %). In the urban area, a significantly higher proportion of female unmarried adolescents at end-line (98 %) had heard about HIV/AIDs from relatives compared to baseline (70 %; *p* < 0.001). The highest proportion of female unmarried adolescents in end-line compared to baseline who learned about HIV/AIDs from relatives in the rural area (100 vs. 67 %). A significantly higher proportion of female unmarried adolescents reported at the end-line compared to baseline in the urban (2 vs. 22 %; *p* < 0.001) and rural (5 vs. 20 %; *p* < 0.001) areas that they had heard about HIV/AIDs from school. A significantly higher proportion of female unmarried adolescents reported at the end-line compared to baseline in the urban (54 vs. 69 %; *p* < 0.001) and rural area (32 vs. 51 %; *p* < 0.001) that HIV can be transmitted by receiving blood from an HIV infected person. A significant proportion of female unmarried adolescents reported at the end-line compared to baseline in the urban (67 vs. 80 %; *p* < 0.001) and rural area (41 vs. 78 %; *p* < 0.001) that using a HIV infected needle or syringe would cause HIV transmission.

**Table 4:** Knowledge on HIV/AIDs, source of information and mode of transmission of HIV/AIDs

Knowledge on HIV/AIDs	Urban Slums Dhaka			Rural Nabiganj		
	Baseline *n* = 800	End-line *n* = 808	*P*-value	Baseline *n* = 833	End-line *n* = 824	*P*-value
Heard about HIV/AIDs	73 %	80 %	***	58 %	49 %	***
Source of information on HIV/AIDs	*n* = 423	*n* = 584	*P*-value	*n* = 174	*n* = 483	*P*-value
Friends	3 %	10 %	***	6 %	4 %	NS
Radio	8 %	4 %	**	14 %	10 %	NS
Television	96 %	95 %	NS	86 %	87 %	NS
Booklet/ magazines/ leaflets/ newspaper	14 %	1 %	***	21 %	2 %	***
School curricula	2 %	22 %	***	5 %	20 %	***
Bill boards/sign boards/posters	3 %	11 %	***	2 %	6 %	***
Public sector	6 %	6 %	NS	2 %	1 %	NS
Relatives	70 %	98 %	***	67 %	100 %	NS
Neighbours	5 %	12 %	***	8 %	6 %	NS
Mode of transmission of HIV/AIDs						
Unprotected sex with HIV-infected person	10 %	9 %	NS	12 %	9 %	*
Receiving blood from HIV-infected person	54 %	69 %	***	32 %	51 %	***
Using needle/syringe infected by HIV	67 %	80 %	***	41 %	78 %	***
HIV-infected mother to children	9 %	11 %	NS	7 %	6 %	NS
Sex with HIV-infected person	10 %	13 %	NS	12 %	11 %	NS
Sex without using condom	3 %	5 %	*	3 %	1 %	**
Sex with sex worker	12 %	12 %	NS	8 %	3 %	***
Sex with multiple sex partners	33 %	28 %	*	30 %	10 %	***

Multiple responses; *p < 0.05; **p < 0.01; ***p < 0.001; *NS* Not significant

Almost 9 % of surveyed female unmarried adolescents knew about STIs in the urban and rural areas (Table 5). In response to a question about whether they knew about some specific names of STI diseases, a significantly higher proportion of female unmarried adolescents in urban area mentioned Syphilis (6 vs. 14 %; *p* < 0.001) and Gonorrhea (5 vs. 9 %; *p* < 0.01) at the end-line compared to baseline. In rural area, 7 % of female unmarried adolescents reported Syphilis/ Gonorrhea at the baseline, and only 8 % of female unmarried adolescent reported Syphilis/ Gonorrhea at the end-line. A significantly higher proportion of female unmarried adolescents reported that they had experienced an STI related symptom, like a sore/ulcer in the genital area (21 vs. 52 %; *p* < 0.001) and excessive per vaginal bleeding (11 vs. 31 %; *p* < 0.001), at the end-line compared to baseline in urban area and significantly higher proportion of female unmarried adolescents reported sore/ulcer in genital area (40 vs. 59 %; *p* < 0.001) and excessive per vaginal bleeding (6 vs. 39 %; *p* < 0.001) in rural area.

**Table 5:** Knowledge on STI and STI symptoms

Knew about types of STI (Unprompted + prompted)	Urban Slums Dhaka			Rural Nabiganj		
	Baseline *n* = 800	End-line *n* = 808	*P*-value	Baseline *n* = 833	End-line *n* = 824	*P*-value
Heard about STI	8 %	9 %	NS	8 %	6 %	NS
Syphilis	6 %	14 %	***	7 %	8 %	NS
Gonorrhea	5 %	9 %	**	7 %	8 %	NS
STI related symptoms						
Sore/ulcer in genital area	21 %	52 %	***	40 %	59 %	***
Excessive per vaginal bleeding	11 %	31 %	***	6 %	39 %	***

*p < 0.05; **p < 0.01; ***p < 0.001; *NS* Not significant

Both at baseline and end-line, 96 % female unmarried adolescents were aware of a healthcare facility in their locality in urban area (Table 6). In rural area, a significantly higher proportion of female unmarried adolescents were aware of a healthcare facility in their locality at the end-line compared to baseline (73 vs. 98 %; *p* < 0.001). A significantly higher proportion of female unmarried adolescents knew at the end-line compared to baseline about government healthcare facilities like: government satellite clinic (4 vs. 26 %; *p* < 0.001) and Expanded Programme on Immunization (EPI) centre (30 vs. 48 %; *p* < 0.001). Knowledge decreased significantly at the end-line compared to baseline about Upazila Health Complex (UHC) and Union Health & Family Welfare Centre (UH&FWC), whereas a significantly higher proportion of adolescents knew at the end-line compared to baseline about non-government healthcare facilities like: NGO static clinic (58 vs. 64 %; *p* < 0.01) & NGO satellite clinic (19 vs. 25 %; *p* < 0.01) and private healthcare facility like: pharmacy (41 vs. 54 %; *p* < 0.001) in urban area.

**Table 6:** Knowledge on types of health facilities in the localities

Knowledge on types of health facilities	Urban Slums Dhaka			Rural Nabiganj		
	Baseline *n* = 800	End-line *n* = 808	*P*-value	Baseline *n* = 833	End-line *n* = 824	*P*-value
Knew about health facility in the locality	96 %	96 %	NS	73 %	98 %	***
Government healthcare facilities						
Government Satellite clinic	6 %	4 %	NS	4 %	26 %	***
UH&FWC	1 %	0.1 %	NS	41 %	30 %	***
Public Hospital	1	0 %	NS	0.3 %	1 %	NS
UHC	0 %	0 %	NS	30 %	9 %	***
EPI Centre	17 %	18 %	NS	30 %	48 %	***
Non-government healthcare facilities						
NGO static clinic	58 %	64 %	**	6 %	2 %	***
NGO satellite clinic	19 %	25 %	**	2 %	2 %	NS
Private healthcare facilities						
Private clinic	21 %	7 %	***	7 %	5 %	NS
Pharmacy	41 %	54 %	***	29 %	26 %	NS

Multiple responses; *p < 0.05; **p < 0.01; ***p < 0.001; *NS* Not significant

## Discussion

The study findings demonstrated a significant increase of knowledge on selected issues regarding reproductive health among female unmarried adolescents. This suggests that the efforts to increase positive knowledge on measures to be taken during menstruation have increased. In addition, adolescents were more knowledgeable at the end-line compared to baseline regarding contraceptive methods namely injectable and condom, mode of transmission of HIV/AIDs and were aware about HIV/AIDs and STI-related symptoms.

Knowledge had significantly improved at the end-line compared to baseline about measures to be taken during menstruation by using clean dry cloth. Findings from a review report on 11 countries’ DHS found that knowledge of a modern method of contraception ranges between 31 and 99 % among adolescents aged 15–19 years [4]. A study in Bangladesh found that 40 % of unmarried adolescents had knowledge on the importance of family planning methods [16]. These study findings are consistent with the end-line findings of this study. In the present study, we found significant changes about knowledge of injectable contraceptives and condoms among female unmarried adolescents at the end-line compared to baseline. Knowledge about long-term contraceptive methods (IUD, Implant and Sterilization) decreased significantly by female unmarried adolescents at the end-line compared to baseline in both areas. The reason might be that peer promoters gave less emphasis on long-term methods when conducting group and individual sessions with female unmarried adolescents. Female unmarried adolescents might be more interested to know about temporary methods.

We found in our present study at the end-line that four-fifths of female unmarried adolescents had heard about HIV/AIDs in the urban slum area, which was higher compared to baseline. The present study found that a higher proportion of female unmarried adolescents at the end-line compared to baseline had learned about HIV/AIDS from relatives and school. School curricula and relatives can play a major role in improving the knowledge of female unmarried adolescents. Overall, female unmarried adolescents’ knowledge about the mode of transmission of HIV/AIDS had improved at the end-line compared to baseline.

The present study reported that overall awareness of STIs was low among surveyed female unmarried adolescents, even at the end-line. The present study found that a large proportion of female unmarried adolescents had improved knowledge about STI-related symptoms at the end-line compared to baseline. As reported by the present study at the end-line, the highest proportion of female unmarried adolescents had heard about a healthcare facility in their locality compared to baseline in the rural area. Their knowledge had increased significantly at the end-line about Government healthcare facilities like: Government satellite clinic and EPI Centre. Knowledge about UHC and UH&FWC decreased significantly at the end-line compared to baseline. The reason might be that female unmarried adolescents remembered and used the nearest and accessible health facilities like: Satellite clinic and EPI centre through the intervention activities, which may be reflected at the end-line survey. In the urban slum area, adolescents had increased knowledge about non-government healthcare facilities and private healthcare facilities like pharmacy. Variations in knowledge of healthcare facilities may be associated with general distribution and availability of health services in Bangladesh. Generally, the Government healthcare facilities are the main source of healthcare services in rural areas, while NGOs and the private healthcare facilities are the most commonly available and utilized facilities in urban areas.

Findings from this study should be viewed in light of several limitations. There was no control area and the present study adopted only pre-post evaluation approach. Data were obtained from only one rural area of one division and one urban area of another division out of seven divisions and the generalizability of these findings to the wider population in Bangladesh or the region is unclear. Additionally, only quantitative analysis was conducted for this study. Use of both quantitative and qualitative methods would have enriched understanding of the context of RH issues and knowledge and perceptions of female unmarried adolescents.

## Conclusion

Adolescents’ knowledge increased in some areas, like: measures to be taken during menstruation, semi-permanent FP methods, HIV/AIDs and healthcare facilities. The intervention increased some reproductive health related knowledge reported in the study. There was no remarkable increase of knowledge on STIs. This intervention can be used to develop appropriate strategies for improving adolescents’ RH-related knowledge, and service delivery so that it is demand-based, effective and replicable in the national program.

## Abbreviations

AIDs: Acquired Immune Deficiency Syndrome
BCC: Behavior Change Communication
BDHS: Bangladesh Demographic Health Survey
CGS: Community Support Group
CI: Confidence Interval
DBRPCP: Demand-Based Reproductive Health Commodity Project
DHS: Demographic Health Survey
EPI: Expanded Programme on Immunization
ERC: Ethical Review Committee
FP: Family Planning
HIV: Human Immunodeficiency Virus
Icddr,b: International Centre for Diarrhoeal Disease Research, Bangladesh
MNCH: Maternal, Neo-natal, and Child Health
MoHFW: Ministry of Health and Family Welfare
NGO: Non-Government Organization
NIPORT: National Institute of Population Research and Training
OR: Operation Research
RH: Reproductive Health
RTM International: Research Training and Management International
STI: Sexually Transmitted Infection
UHC: Upazila Health Complex
UH&FWC: Union Health & Family Welfare Centre
